# Systemic and individual factors in the buprenorphine treatment-seeking process: a qualitative study

**DOI:** 10.1186/s13011-016-0085-y

**Published:** 2017-01-11

**Authors:** Valerie M. Hewell, Angel R. Vasquez, Inna D. Rivkin

**Affiliations:** Department of Psychology, University of Alaska Fairbanks, P.O. Box 756480, AK 99775 Fairbanks, USA

**Keywords:** Medication-assisted treatment, Qualitative research, Opioid use, Treatment-seeking, Policy

## Abstract

**Background:**

Opioid use is a significant problem in Alaska. Medication-assisted treatment for opioid use, including buprenorphine, reduces withdrawal symptoms and the harm associated with opioid abuse. Understanding consumers’ treatment-seeking process is important for addressing barriers to treatment, facilitating effective service utilization, and informing policy.

**Methods:**

To understand treatment-seeking behavior, we examined the attitudes, perceptions, and knowledge of those who would benefit from the medication-assisted treatment (MAT) buprenorphine. Qualitative data from 2 focus groups (each including 4 participants) and 3 in-depth interviews with people who have used or considered using buprenorphine in treatment for an opioid use disorder were analyzed using grounded theory and directed content analysis approaches.

**Results:**

Key findings suggest that individual (withdrawal process, individual motivation) and systemic (sociocultural, political, societal values) factors frame the treatment seeking process. Participants’ progress on the treatment-seeking road was affected by models of addiction and MAT, which related to facilitators and barriers encountered in seeking treatment (e.g. support, resources, treatment structure). These factors shaped the longer-term road to recovery, which was seen as on ongoing process.

**Conclusions:**

The findings of this study suggest it is crucial for interventionists to take a contextual approach that considers individual and systemic factors involved in opioid addiction, treatment, and recovery. This study highlights ways policy makers and treatment providers can address the barriers consumers face in their treatment-seeking process in order to increase treatment access.

## Background

Opioid use disorder involves continued misuse of opioids despite recurrent problems occurring from use [1]. Due to the sizable incidences of nonmedical prescription opioid initiation [2] and the harm associated with opioid use including rising rates of overdose [3], there is alleged to be a national opioid epidemic in the United States [3–5]. Opioid misuse is also a noteworthy problem in the state of Alaska, which consistently ranks in the top ten states of the nation for illicit drug dependence [6]. Moreover, fatal drug overdose rates, the majority of which include prescription drugs, increased by 55% between 1999 and 2010 in Alaska [7]. Particularly, in Alaska, the unmet treatment needs of citizens with drug dependence have consistently registered above the national average [6]. Moreover, disparities have been found in the services patients with co-occurring disorders receive, with those presenting for substance abuse, as opposed to mental health, treatment receiving less mental health care despite having the same diagnoses [8]. The rapidly rising rates of opioid misuse and the harm associated with nonmedical prescription opioid use compound the foregoing unmet needs of people who would benefit from behavioral health services.

Medication-assisted treatment (MAT) for opioid use disorders, which use psychotropic drugs usually combined with psychosocial treatment, has been empirically indicated to reduce withdrawal symptoms, opioid abuse, and the harm associated with opioid use [9–14]. For opioid use disorders, MAT has frequently been associated with the long-time “standard” replacement therapy: methadone [9]. While methadone is an empirically validated medication that has illustrated efficacy in increasing the viability of recovery by improving treatment retention and reducing opioid use compared to non-pharmacological alternatives in people with opioid dependence [14], it may not be the treatment of choice for some patients.

In 2002, the FDA approved buprenorphine as a new MAT for opioid use disorders under the Drug Addiction Treatment Act of 2000 [15]. Buprenorphine (Subutex®) and the buprenorphine/naloxone combination (Suboxone®) are established as effective, safe, and well-tolerated pharmaceutical treatments [10, 11] that increase patient engagement and retention while reducing symptoms associated with opioid use disorders [11, 13]. Compared with methadone (which must be dispensed at designated opioid treatment programs), buprenorphine is prescribed by a qualified general physician in an office-based setting and may increase access to treatment [13]. Although the effectiveness of buprenorphine is established, less is known about the treatment-seeking behavior of those who would benefit from buprenorphine treatment.

### Project rationale

It is important to understand the attitudes and perceptions of those who would benefit from treatment (now referred to as consumers) as these factors influence the utilization of services. Studies assessing treatment-seeking tendencies for addictive disorders highlight barriers including stigma, misattunement to patient needs (i.e., lack of gender-specific services), and financial cost [16, 17]. While few qualitative studies have explored what treatment consumers identify as being relevant to their treatment-seeking process, one such qualitative study explored motivations for seeking treatment among a broad array of substance users [18]. Such motivations included dissatisfaction with oneself, influences of friends, family, and spiritual interventions. As one systematic literature review accentuated, the majority of the research on individuals’ reasons for entering substance misuse treatment has been quantitative, which regrettably deemphasizes the patient’s perspective [19]. As such, the use of qualitative methodology is recommended for extending such understanding [19]. The current study explores consumer perspectives of the MAT-seeking experience using qualitative methodology.

Understanding treatment seeking from the consumer perspective has implications for policy. For instance, restrictive federally imposed limits on the number of buprenorphine patients a provider can treat may serve as a barrier for receiving services [15], particularly in communities with limited provider availability. With all of this in mind, there remains a need to understand the subjective treatment-seeking process of consumers with self-identified opioid use disorder who would benefit from MAT. Such an understanding can help policy leaders and interventionists make decisions that facilitate, rather than stymie, individual treatment seeking in addition to illustrating how existing policies and protocols affect the lives of consumers.

## Method

This research was based on a larger mixed-methods study that explored how both treatment providers and potential treatment consumers understand MAT. The aim of the current project was to explore what factors influenced Alaskan MAT consumers (including those who sought or considered using MAT) treatment-seeking process.

### Community involvement and ethical considerations

The study was conducted in Fairbanks, a city of about 32,000 people in the Interior region of Alaska [20]. Although this population meets criteria for an urban area according to the United States Census Bureau [21], the Fairbanks and Interior region has many qualities of rural life (e.g., higher rates of substance abuse, limited access to health care, travel barriers, and stigma associated with behavioral health services) [22]. This project utilized community-based participatory research principles including shared ownership, community analysis of social problems, a strength-based and collaborative approach, a focus on action, and an iterative process [23, 24]. As such, it operated under the guidance of Turning Point Counseling Services (a local private counseling and substance abuse center), the Alaska Advisory Board on Alcoholism and Drug Abuse, stakeholders in the community, and MAT professionals. It was approved by the University of Alaska Fairbanks Institutional Review Board (523384–9) and funded by the Alaska Mental Health Trust Authority.

### Participants

The study sample included people 18 years or older living in Alaska who: (1) met criteria for an opioid use disorder at some point in their life, and (2) had been impacted by medication-assisted service delivery (e.g., received services, been denied of services, encountered barriers to obtaining services, or avoided seeking services). Exclusion criteria included individuals that were actively suicidal, experiencing psychosis, or who directly received services from the researchers who collected data.

Focus groups and semi-structured interviews explored participants’ perceptions relevant to MAT. There were a total of 11 participants; the majority were female (64%, *n* = 7). Nine participants had received MAT at some point in their lives, seven had been or were currently in a methadone treatment program, and two had been in a buprenorphine treatment program.

The first focus group was conducted with four patients who were currently engaged in MAT. Subsequently, three interviews were conducted with individuals who were opioid-free at the time of the interview. Finally, a second focus group, which included four individuals in recovery who were receiving support from a mutual self-help group, was conducted. All study participants had considered using buprenorphine treatment at some point in their recovery.

### Procedure

Recruitment strategies included word-of-mouth, flyer, and networking techniques. Individuals who participated in the study were asked if they knew others who might be interested in participating. This snowball sampling procedure was utilized to build relationships, trust, and gain entry into the community. Individuals were screened for study criteria and, after obtaining written informed consent, all interviews and focus groups were audio-recorded. Upon completion of data collection, participants were screened for distress, debriefed, thanked, and compensated with $30 gift certificates.

Participants were queried about the following: (1) experiences seeking treatment including barriers to entering and remaining in treatment (e.g., “Some people report that they had a hard time receiving medication-assisted services for a number of reasons. When you were seeking treatment, did you experience any barriers?”) (2) treatment preferences (e.g., “what would the ideal program consist of?”), (3) success in MAT (e.g., “What successes have you experienced after engaging in medication-assisted treatment?”), and (4) beliefs about the cause of addiction (e.g., “What are the most important factors that contribute to your addiction?”). Participants were also asked about perceptions and attitudes related to MAT for themselves and for others (e.g., “What do you think about buprenorphine?”, and “What attitudes do you think community members have toward Medication-assisted treatment?”).

Following the grounded theory techniques of theoretical sampling [25], data collection was informed by participant responses. After each interview or focus group was conducted, researchers discussed impressions, which informed subsequent prompts. After each phase of data collection, raw data in the form of interview and focus group audio recordings was transcribed and input into Nvivo^10^ software.

### Data analysis

Using grounded theory, data were coded using open, axial, and selective coding techniques [25]. The researchers evaluated their biases and mitigated these by consulting with an expert in qualitative analysis that had limited knowledge of opioid use disorders.

The first two authors collectively coded each transcript into the major content domains, developed an initial codebook for each domain, and began to code the domains using the initial codebook. After agreeing on the dependability of the codebook, they independently coded the domains and routinely checked for agreement. Discrepancies were collaboratively discussed. These strategies served to enhance reliability and protect against coder drift.

## Results

**Fig. 1:**
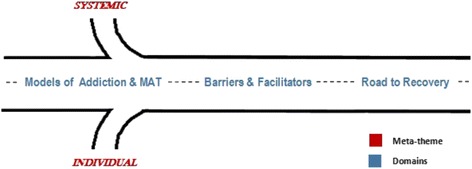
Treatment-seeking road

The conceptual model emerging from the analyses illustrates an overarching meta-theme (individual and systemic factors) as well as domains that influenced the treatment-seeking process (see: Fig. 1). The model is best described as a highway with on-ramps and exits that can keep individuals on a path of opioid misuse or facilitate treatment seeking, which ultimately leads to the road to recovery.

### Individual and systemic factors

Participant stories highlighted the role of individual and systemic factors, which contextually frame the treatment-seeking road as a meta-theme. These factors influenced an individual being able to enter, or having to exit from, their treatment-seeking road along the three domains (described below).

Individual factors included intrinsic attitudes, beliefs, values, and motivations in seeking and completing treatment. Systemic factors included broader social support, family, treatment, public policy, and culture. The individual and systemic influences interacted bidirectionally along each individual’s unique treatment-seeking road. For instance, one participant recalled, “once I started going and hearing from others, hearing what the program means, going to meetings, I started experiencing strength and hope…I wanted what they had…I’m sick and tired of being sick and tired.” In this example, the participant had internal factors (being “sick and tired of being sick and tired”) that interacted with external influences (supportive others engaged in treatment), and influenced their treatment-seeking road through social learning (“experiencing strength and hope”). This meta-theme is present throughout the three key domains as they relate to the consumer’s experience before and during treatment, and in recovery.

### Key domains

While the meta-theme encompasses broad elements framing participants’ experiences, three key domains represent qualities that affected how participants navigated their treatment-seeking road, including: (a) *models of addiction and MAT*, (b) *barriers and facilitators*, and, (c) *the road to recovery. Models of addiction and MAT* included implicit factors that inform the participants’ treatment-seeking process. *Facilitators and barriers* included experiences that made it easier or harder to obtain treatment. All of these constructs shaped the participants’ *road to recovery*, which was primarily described as a process that happens later in, or after, treatment.

This process is represented as a road in Fig. 1 to illustrate the progressive nature of treatment-seeking. However, just like merging lanes on a highway, degrees of overlap along the treatment-seeking road exist. For instance, participants might describe barriers that are based in *models of addiction and MAT*, or they might describe facilitators that influence their *road to recovery*.

#### Models of addiction and MAT

Participants shared their lived experiences, beliefs, and understandings related to addiction and MAT, which established internal models. These models operated as inherent frameworks that influenced their treatment-seeking process. Specifically, participants described how they understood or explained the cause of addiction, beliefs about getting clean, and the withdrawal process. One participant highlighted the individual and systemic influences involved in his understanding of addiction:

*For me there are both individual and social factors. Individually… genetically, I come from a long line of people who are addicted to substances. Socially, culturally, (substance abuse in) my neighborhood where I grew up was culturally appropriate. A man drank; got loaded; it was actually a rite of passage. And I think after that particular point, it kind of woke up the dragon biologically and almost created a downward spiral in a lot of scenarios. Physically, emotionally, and the fact that I came from an area that I guess you could say struggled. The whole community struggled with addiction so it was kind of like this acceptance of everybody kind of like abus(ing). That was a contributing factor. It was a way of coping with things—to medicate with substances.*

Explanatory models of addiction such as the example above informed participants’ beliefs about getting clean. For instance, believing that addiction is caused by biological and contextual factors may lead someone to believe that biological and social components must be addressed in order to recover (e.g., by reducing physiological cravings and building social support for recovery). These explanatory models had implications for treatment, as many participants in this study believed recovery was a lifelong process.

Additionally, withdrawal was described as a difficult aspect of addiction that sometimes led people to go back to misusing opioids. As this participant illustrated: “(Withdrawal) was physical, mental, emotional, spiritual. I was a disaster for like…I think I made it…four days, and then I went back.” Participants reported withdrawal made it challenging to quit or stay off opioids without support. As one participant noted, “So I had to come off of it cold turkey, and it was a terrible, terrible experience, so I just went back to heroin.” As such, participants described MAT as being helpful in decreasing withdrawal symptoms, which allowed it to be used as a “stepping stone” to recovery.

In addition to their models of addiction, participants discussed their models of MAT. This included perceptions of how others feel about MAT, and participants’ own implicit beliefs about MAT. Participants primarily perceived others as having negative views, including stigma and judgment, about MAT. Negative societal views, including the belief that people on MAT were still addicts and not in the recovery process, interfered with participants’ progress along the treatment-seeking road. Participants believed that these views were communicated overtly (e.g., transmitted through the media) and covertly (e.g., through misassumptions acquaintances had). For example, one participant stated:

*I hear it all the time: ‘you’re not sober’. And it really hurts my feelings because I worked hard…from where I was to where I (am) now…I got my own place, I got my disability, I got everything on track. And all she said was, ‘you’re still not sober’.*

Nevertheless, participants had varying personal beliefs about MAT, including believing it was a good aid in the recovery process, thinking it was trading one drug for another, and having negative emotions associated with MAT. For instance, one participant stated:

*Looking back on it, (buprenorphine) was my miracle drug because I know I couldn’t do it without that. That first three months was so painful, I felt I was being ripped apart by wolves, the cravings were so intense.*

However, another noted, “I guess I felt like, looking back on that, I feel like I put like a Band-Aid® on my gunshot wound.” Regardless of whether individuals felt that MAT was beneficial or detrimental, all participants discussed feeling that MAT alone was not enough and should be paired with support or treatment. In terms of recovery, one participant noted the importance of an integrated buprenorphine program:

*It took me a long time to realize how important [buprenorphine] was to my recovery—it was that stepping stone from before and after. I wouldn’t be [here] if it wasn’t for the in-between with the […] program.*

#### Facilitators and barriers to seeking treatment

Participants discussed four facilitators and barriers including attitudes, support (or the lack thereof) in treatment, resources, and the structure and context of treatment. These facilitators and barriers shaped participants’ attempts to seek MAT.

##### Attitudes

Certain attitudes, including social and internalized stigma (for utilizing MAT) that were affected by societal values, and a personal decision to change emerged from participants’ stories as being relevant to their ability to enter treatment. These attitudes, which served as facilitators or barriers to treatment, were necessarily influenced by the implicit models discussed above. Social stigma, including negative public beliefs about addiction, presented a roadblock to seeking MAT by affecting receptivity to treatment. One participant noted:

*“People are like, ‘drug addicts are evil. Evil junkies, evil!’ So I don't even think people are really educated on drugs, much less treatments or [buprenorphine]…that is where every goes wrong…because most people don’t want to get help or talk about it cause you’re [assumed to be] evil.”*

This example depicts how societal stigma stems from a lack of health literacy about MAT. This societal stigma can stymie consumers’ reaching out and getting into MAT, even if they believe it is effective.

Participants highlighted the need to change negative attitudes about addiction, substance abuse treatment, and MAT on a systemic level. One participant suggested, “creating a culture that is conducive and attractive for people in recovery for all different manifestations of recovery whether it’s medically-assisted or abstinence, whatever.”

Relatedly, values that spur from our “Puritan Society” could contribute to societal or internalized stigma. Societal stigma, such as the belief that those who have an addictive disorder have a poor character or the de-emphasis on the biological contributions on addiction, were described as barriers to seeing MAT as an acceptable option for some. Additionally, an individual’s culture (e.g., ethnicity, socio-economic background, social ecological history) influenced his or her treatment-seeking experience. One participant stated:

*I think being Alaska Native and having addiction and trying to get help, there’s a huge stigma to that. It’s kind of like, you’re a shame. It’s like very shameful. We’re very prideful people, you know? It’s a lot harder for people to get clean.*

This example accentuates the importance of considering how an individual’s cultural context impacts the societal stigma they face and the obstacles they encounter in seeking treatment. It also illustrates how societal stigma may be internalized, thereby effecting consumers’ feelings about themselves as they try to access treatment, including the experience of shame.

While social and internalized stigma could make it difficult to seek treatment, personal decision to change accelerated participant progress along the treatment-seeking road. For instance, one participant noted, “you have to want it,” and another accentuated that staying clean was the most important thing in his life: “I want it more than anything. Stay clean first. That’s about it. I have nothing without it.”

##### Support

Some participants believed their therapists’ support, unconditional positive regard, and collaborative treatment environment are what got them through treatment challenges. One participant discussed the role of a compassionate and collaborative treatment environment: “What I’ve seen be very successful is just compassion and like, ‘let’s do this. Like let’s figure it out together.’”

Others felt that treatment was nontherapeutic and unglamorous, and that providers were unhelpful, “jaded”, and/or lacked health literacy, which presented significant barriers to seeking and staying in treatment. Likewise, another participant discussed how stigma and the lack of support in the treatment environment could negatively impact recovery: “Well, I guess if you’re on [buprenorphine] and you go to meetings and you just hear whisperings and there was stigma …You don't want to go to a meeting where it’s not unconditional love.”

Support and connection across different systems (e.g., social, familial) was also described as a contributing factor in addiction, treatment seeking, and recovery. One participant noted:

*When you’re using, you have a tribe. When in recovery and I go to NA, I have a tribe. When I relapsed, what made me do that was I betrayed my using tribe…People are necessary for recovery. It’s necessary for me. Connection. That’s it…it’s saving my life.*

This powerful quote illustrates how support and connection affect the entire treatment-seeking process, from addiction to relapse to recovery. Clearly, support and connection have an important role in why participants do or do not seek MAT.

##### Resources

The lack of resources, including health literacy, access/availability, and financial resources, were primarily discussed as barriers to MAT. For instance, one participant highlighted the detrimental influence of limited treatment availability:

*“there’s a very narrow window of opportunity in somebody’s addiction to get in (treatment) so when they finally decide, okay I think I need something, and then there’s nothing available, then that window has passed and you have to wait for the next one.”*

This acute description demonstrates the need for availability and swift action on the part of treatment facilities. Additionally, finances served as a barrier for many as illustrated by this participant: “I think the financial aspect is one of people’s biggest fears. Because they don’t have money. And even the willingness to make payment plans is more incentive for people to get help.” As a result of the lack of treatment availability and financial barriers, many participants were left feeling hopeless and getting high, having to travel long distances in dangerous circumstances to seek treatment, or committing a crime to go to jail to “get clean.”

Finally, participants attributed stigma and misassumptions about MAT to a lack of health literacy: “There’s no knowledge about it, people just don’t understand it. And they’re not going to unless they’re told.” Nevertheless, our analysis revealed that education alone is not enough. One participant stated, “Education may not always be the answer…I do believe information, education…those are things that for all providers might be helpful; not so much if the person presenting it has their own biases.” In other words, it is important to increase health literacy while also addressing implicit and systemic stigma about MAT. Undoubtedly, access to both physical and informational resources are important for progression along the treatment-seeking road.

##### Treatment structure

In regards to treatment structure, participants discussed a strict treatment structure as beneficial in the beginning of treatment, while noting that more autonomous functioning can facilitate recovery later in treatment. In this way, some participants implied that MAT could itself become a source of oppression and restriction that kept them locked in through an “umbilical cord”. Alternatively, MAT that offered more freedom and flexibility, especially when the consumer was stable, was described as having the ability to foster strength. As this participant noted: “I honestly, first time in my life, took it like I was supposed to. Took it like the bottle told me to. And it was kind of a liberating experience. To be in control and in possession of something like that.” This speaks to the importance of matching the treatment structure with where consumers are developmentally.

Overall, the various treatment facilitators and barriers interacted with each other to influence the road to recovery. For example, barriers such as stigma and access to care were especially difficult to overcome for consumers with less support (e.g., family, recovery tribe). On the other hand, motivation to change was a primary source of strength, inspiring individuals to seek treatment even in the face of barriers.

#### Road to recovery

In addition to *models of addiction and MAT* and *barriers and facilitators*, participants discussed their experiences and expectancies later in treatment, or their *road to recovery*. This included reaching a critical mass point, internal factors (e.g., taking personal responsibility), contextual factors (e.g., having connection and support), and living a program of recovery (e.g., having investment in the process).

Reaching a “critical mass point” was key in participants’ transition toward recovery. This was characterized as dramatic moments that prompted participants to change including having a relapse, hitting rock bottom, perceiving that they had a choice to make changes, “being sick and tired of being sick and tired”, and realizing that misusing opioids was no longer an effective coping mechanism. After the critical mass point was reached, both internal and external factors helped people move toward recovery.

Internal factors seen as necessary to longer-term “recovery” included will, dedication, clarity of values, and spirituality. These factors were often described as influential in helping participants overcome adversity. In the following example, will and clarity of values helped an individual persist through difficult situations to remain in the living program of recovery after treatment:

*I think there are people who are strong enough to get through (the stigma). I probably wouldn’t give a shit, I want to get clean you know? I know what I want. But other people are not like that. They’re not that strong willed or minded.*

In addition to will, motivation and believing in oneself were described as important. For instance, one participant stated, “I feel like you can’t push somebody. You got to come to it on your own.” Another participant pointed out, “You have to change. That was a huge epiphany for me: that I’m the problem.”

External factors (e.g., a supportive clinician, positive treatment structure, social learning through peers) helped participants continue moving forward on their road to recovery. One participant described how her dedication to her family kept her “sober”:

*Every time I would think of feeling like I wanted to use again, I would just think about losing [my kids]. I would never, ever want to lose them or leave them or do anything—and it’s a big one for me. It’s kept me sober for the last 4 years.*

Even so, participants did not discuss recovery as a permanent point, but they believed it was important to have a living program of recovery. They generally believed this living program of recovery was an individualized process that is different for every person, but likely included an investment to themselves to stick to treatment, changes in thought processes, using MAT as a stepping-stone toward recovery, and going through a process of change that was not a “quick fix.” Participants discussed the progress they had made, and many were able to look back and notice how they progressed. For instance, one participant recalled, “If you really do look at everybody in here and where they were compared to where they are now, like most of us have gotten back on track, we all have our kids, gone back to school.” Continuing to progress was also a component of the living program of recovery. As this person noted, “I can’t stop. It never stops, the disease doesn’t go away.”

## Discussion

The current study is one of the few studies to examine the attitudes and perceptions of consumers who may benefit from the MAT, buprenorphine. It illustrates the individual and systemic factors that affect the treatment-seeking process and elucidates the importance of contextual factors, such as stigma, access to resources, and support. Ecological systems theory [26] provides a useful framework for conceptualizing this study’s findings. It posits that each individual exists within a nest of systems that contextualizes and influences individuals’ behaviors [26].

In our study, each person’s addiction, treatment-seeking process, and recovery can be framed within the nested systems. These systems include macrosystem (e.g., cultural norms, protestant values, abstinence-only orientation), exosystem (e.g., mass media as a source of stigma, public policy), mesosystem (e.g., insurance, communication between providers), microsystem (e.g., consequences of drug use on family, employment, social supports), and individual (e.g., motivation to change, willpower).

The important role ecological factors play in the development, maintenance, and treatment of substance abuse is well established [27] and is also considered in the National Institute of Drug Abuse’s [NIDA] principles of effective treatment [28], which includes consideration of treatment and biopsychosocial individual factors. Results from the present study expand upon NIDA’s principles for effective treatment by considering broader ecological influences including societal attitudes, cultural norms, access to resources, and policy that influences how treatment is delivered.

The role individual and systemic factors play in MAT seeking found in this study corroborates with the construct of recovery capital [29–31]. The construct of recovery capital proposes that the accumulation of internal and external resources affect one’s ability to recover from addiction. It is suggested that professionals working with people in treatment for addiction can facilitate recovery on three levels of recovery capital: personal recovery capital, family/social recovery capital, and community recovery capital [31]. This study’s findings demonstrate similar constructs are relevant to MAT seeking while revealing the interactional relationship of these resources.

For example, macrosystem, exosystem, and mesosystem (e.g., cultural assumptions, policy and funding) influences frequently served as barriers, whereas participants’ individual and microsystem resources (e.g., motivation to change, having support from family and treatment programs) more frequently served as facilitators to treatment and recovery. As such, policy makers might take these findings into consideration by creating policy and allocating funds that support microsystems from the bottom-up, rather than dictating the operation of treatment from the top-down. This may include providing funding for rural areas or in areas that are affected by opioid misuse, or allowing treatment programs to decide their own treatment operations (including the number of patients a provider can serve) as opposed to having to meet a federally imposed standard. Also, treatment practitioners may want to focus on individual strengths that can serve as personal resources for recovery.

Importantly, the source of power affecting MAT seeking could originate in any system and could change overtime. For instance, someone may originally seek MAT because of their children (microsystem level influence) yet remain in treatment because of their children and their own individual motivation (microsystem and individual level influences). Likewise, practitioners should keep in mind that the source and degree of motivation fluctuate, and refine interventions accordingly, flexibly drawing on shifting sources of recovery capital.

Participants in our study discussed willpower, believing in one’s ability to change, and motivation to change as individual factors important for seeking treatment and maintaining recovery. Similarly, self-efficacy [32–34], defined as confidence in one’s ability to do something (e.g., be abstinent or not use), and perceived locus of control [35], defined as the belief that change is within one’s power, play important roles in overcoming substance use disorders. Participants viewed buprenorphine as a tool for gaining such control – a stepping-stone to recovery. This is consistent with Miller and Rollnick’s [36] work positing that three conditions are necessary for change: being ready, willing, and able. For some participants, MAT was a tool in their process of cultivating readiness (helped them prioritize and feel capable of quitting), willingness (helped them believe that change is important), and ability (helped them manage withdrawal symptoms, which built confidence and investment to change).

In this study, specific individual factors, including self-efficacy and motivation to change, facilitate MAT seeking. Thus, interventionists should target their efforts with self-efficacy and motivation to change in mind. Perhaps a practitioner could work to increase self-efficacy by taking a strength-based approach and cater treatment to the consumer’s motivation to change by using a motivational interviewing approach. Additionally, a community psychologist could emphasize these individual strengths when developing community interventions.

Participants in this study also emphasized the importance of contextual factors in treatment-seeking decisions, including support from family, others in treatment, treatment providers, and the broader community and context. Family history and social support are recognized as important external factors that affect addiction and treatment [35]. In one study with people with opioid use disorders, a lower quality of social support related to higher perceived stress, which in turn was associated with greater opioid misuse [37]. While the negative consequences of poor social support was mentioned by our participants, they primarily emphasized positive aspects of social interactions, including the way compassion and positive social support facilitated their treatment-seeking process.

Because some factors of the community and cultural context could serve as facilitators or barriers, it is important for interventionists to take this into account when working with people in MAT. For instance, therapists should consider the individual in their cultural context. Additionally, societal stigma should be addressed by considering the cultural context in which it is nested.

Health literacy and the treatment environment also influenced participants’ ability to get what they needed out of treatment. Many participants described being “uninformed” when seeking treatment. While health literacy can combat some barriers, societal stigma must also be addressed by challenging attitudes. Thus macrosystem interventions should inform those who would benefit from MAT about treatment options and educate people globally about MAT to challenge attitudes and fight societal stigma.

While MAT facilitated recovery for some participants, it was also described as a barrier to furthering recovery when consumers felt trapped in the system. This corresponds with findings from a previous qualitative study where participants described methadone as “liquid handcuffs” that prevented them from actualizing recovery [38]. Thankfully, a supportive and collaborative therapist and treatment structure were described as significant facilitators to staying in treatment and maintaining recovery. Similarly, the therapeutic alliance, which is patient-therapist agreement on goals, task, and the bond [39] has been shown to improve outcomes for those with opioid dependence [40]. Thus, providers and treatment facilities should cultivate a supportive treatment environment and encourage consumer autonomy.

Finally, there are unique challenges that rural citizens face due to distinctive cultural and geographical context, including higher rates of substance abuse, limited access to health care, travel barriers, and stigma associated with behavioral health services [22]. The participants of this study accentuated the geographical and availability barriers in Alaska. Even when participants are motivated to change, long waiting lists and limited treatment availability can close a valuable window of opportunity. In rural settings with limited providers and geographical challenges that make it difficult to obtain treatment elsewhere, federally imposed caps on patient limits [15] for buprenorphine providers can be seen as an even bigger barrier to treatment. Policy makers may consider advocating for making exceptions to the provider limit on patients, particularly in rural areas.

These barriers to access have significant practical implications for society because, while substance abuse may commonly be assumed to be an individual disease, its social impacts on health and judicial systems exceed $510 billion annually [2]. MAT is only accessible to a limited number of individuals who would benefit from treatment [2], and contextual factors (including socioeconomic status and rural location) affect treatment access [22].

### Strengths, limitations, and future directions

A major limitation of this study is that it included a small sample. While we used snowball-sampling procedures to attempt to reach a broad range of those who would benefit from MAT, most participants were aware of some treatment services, and we were limited in our ability to reach individuals who do not access treatment services. Thus, this convenience sample may not be representative of those who are very limited in resources. The limited sample warrants caution for generalizing results. Additionally, our model is derived from a sample that has contextual factors that may not generalize directly to other communities. Therefore, specific influences that affect MAT treatment seeking may vary. Future research exploring MAT treatment seeking in underserved communities should attempt to obtain larger, diverse and representative samples of those who would benefit from MAT.

However, like another qualitative study [41], the sample size was sufficient for illuminating consumers’ central themes and concerns related to the research questions of this study, and therefore reaching data saturation. Additionally, our study provides an in-depth perspective of MAT treatment seeking, using participants’ own words to illustrate their struggles and successes, and offering treatment providers and policy makers insight regarding participants’ lived experiences through their own eyes [42]. It integrates a community-based approach in a real-world setting, which increases validity through drawing on community expertise, builds empowerment, and facilitates the utilization of findings [43–45].

While research has explored attitudes towards methadone, this is one of the few studies to qualitatively examine the perceptions of those who would benefit from buprenorphine treatment. Although this treatment has demonstrated efficacy [9–14], the full potential of services cannot be actualized unless the consumer is at the forefront of treatment. Moreover, these consumers’ perspectives may inform considerations for buprenorphine policy. Future research can extend on the current project by investigating the difference between methadone and buprenorphine in facilitating recovery, the meaning of success in buprenorphine treatment, and the role of systemic barriers, including funding and systemic support of buprenorphine MAT.

## Conclusion

The current study highlights that addressing the interrelated individual and contextual factors affecting treatment-seeking is critical. Further, there are discrepancies between what is needed for optimal progress on treatment-seeking road and the political and contextual barriers consumers may face in obtaining timely treatment that addresses their needs. As such, interventionists should recognize individual strengths while prioritizing the cultural milieu when advising clinical and community change efforts to improve the delivery of MAT and buprenorphine services. In doing so, health literacy, compassion, and understanding should be increased and disseminated across the different systems (i.e., policy makers, general public, frontline service delivery professionals, consumers) while combatting negative and misinformed beliefs that perpetuate stigma. Finally, our findings echo that it is important to increase access to MAT programs that integrate medication with psychosocial components in substance use disorder treatment [13, 46].

## Abbreviations

FDA: Food and Drug Administration
MAT: Medication-assisted treatment
NIDA: National Institute on Drug Abuse
